# De-aberration for noninvasive transcranial photoacoustic computed tomography through an adult human skull

**DOI:** 10.1038/s42005-026-02545-3

**Published:** 2026-02-14

**Authors:** Yousuf Aborahama, Karteekeya Sastry, Manxiu Cui, Yang Zhang, Yilin Luo, Rui Cao, Geng Ku, Jigmi Basumatary, Junhao Zhu, Siying Kong, Lihong V. Wang

**Affiliations:** 1 Caltech Optical Imaging Laboratory, Andrew and Peggy Cherng Department of Medical Engineering, California Institute of Technology, 1200 East California Boulevard, Pasadena, CA 91125 USA; 2 Caltech Optical Imaging Laboratory, Department of Electrical Engineering, California Institute of Technology, 1200 East California Boulevard, Pasadena, CA 91125 USA

**Keywords:** Photoacoustics, Acoustics

## Abstract

Noninvasive transcranial photoacoustic computed tomography (PACT) of the human brain, despite its clinical potential as a complementary technology to functional MRI, remains impeded by the acoustic distortion induced by the human skull. The distortion, which is attributed to the markedly different material properties of the skull relative to soft tissue, results in heavily aberrated PACT images. Herein, we report an experimental demonstration of the de-aberration of PACT images through an ex-vivo adult human skull using a homogeneous elastic model for the skull. Using only the geometry, position, and orientation of the skull, obtained from adjunct imaging data and fiducial markers, we faithfully de-aberrate the PACT images of light-absorbing phantoms acquired through an ex-vivo human skull for different levels of phantom complexity and positions. We also demonstrate the generality of our results by attaining a similar extent of de-aberration through a second ex-vivo human skull. Our work addresses the longstanding challenge of skull-induced aberrations in transcranial PACT and advances the field towards unlocking the full potential of transcranial human brain PACT.

## Abstract (web summary)

Noninvasive transcranial photoacoustic computed tomography (PACT) of the human brain remains impeded by the acoustic distortion induced by the skull. Here, using a homogeneous elastic skull model, the authors de-aberrate the PACT images of light-absorbing phantoms acquired through an ex-vivo human skull for different levels of phantom complexity.

## Introduction

Photoacoustic computed tomography (PACT) is an emerging technique that enables molecule-specific optical absorption imaging at centimeter-scale depth^1^. It is based on the generation of acoustic waves upon the absorption of light by tissue, namely, the photoacoustic (PA) effect^2^. Owing to the approximately homogeneous acoustic properties of soft tissues, the generated initial pressure map in tissue, which is proportional to the optical absorption coefficient distribution, can be readily reconstructed from the recorded PA signals around the target using the universal backprojection (UBP) method^3^ or iterative model-based methods^4,5^. Hence, PACT has been used for imaging several parts of the human body, such as the breast^6–8^, extremities^9,10^, and neck^11,12^.

PACT of the human brain has also been investigated^13,14^ as it offers distinct advantages over the widely used functional magnetic resonance imaging (fMRI). PACT relies on the strong optical absorption of hemoglobin relative to the surrounding tissue. By leveraging the distinct spectral signatures of hemoglobin, PACT is directly sensitive to hemodynamic variations associated with both oxy- and deoxy-hemoglobin. This offers a comprehensive hemodynamic contrast distinct from standard BOLD fMRI, which relies primarily on magnetic susceptibility changes driven by deoxy-hemoglobin and is therefore indirectly sensitive to only one of the two forms of hemoglobin. It is more portable, more space-efficient, more open, less noisy, less expensive, and cheaper to maintain than fMRI. Further, due to its magnet-free operation, it can be used for subjects with implants that are incompatible with fMRI. Recently, the PACT of human brain vasculature and function was demonstrated in the special population of hemicraniectomy patients in whom a section of the skull is surgically removed to release elevated brain pressure^15^. It was validated with blood oxygen level-dependent (BOLD) fMRI at the highest clinically allowed magnetic field strength, thus demonstrating its clinical potential. However, for this technology to be applicable to the general adult human population, the challenge posed by the human skull needs to be overcome.

The mechanical properties of the human skull differ significantly from those of soft tissue^13^. Unlike soft tissue, the skull supports both compression and shear waves, thus resulting in mode conversion at the soft tissue-skull interface^16^. The average compression wave speed and the density of the skull are also approximately twice that of soft tissue^17^, thus resulting in an impedance mismatch at the skull-tissue interfaces. Moreover, the skull is a dispersive medium and induces frequency-dependent attenuation. Thus, the waves passing through the skull are severely distorted, which causes the resulting PA images, reconstructed using conventional image reconstruction methods (e.g., UBP), which assume homogeneous tissue properties, to be highly aberrated. This distortion is also detrimental to human brain functional imaging since it degrades the strength of the detected functional signals and the accuracy of localization of functional regions, as shown in this preliminary study^18^. To overcome this problem, the properties of the skull need to be incorporated into the image reconstruction method. Notably, the skull also induces light attenuation in the near-infrared wavelength range (700–1100 nm) primarily due to scattering^16,19^. At 1064 nm, a 4 mm-thick skull bone induces ~50% optical attenuation^13,15^.

Despite numerous attempts over the last two decades^20–28^, a conclusive experimental demonstration of de-aberration in transcranial PA images remains elusive. A layered back-projection method^24^ was developed for transcranial PACT by extending UBP to a piecewise homogeneous medium. However, this method cannot account for reverberations within the skull and reflections of waves arising from absorbers outside the skull. Further, it did not accurately model the brain-skull and skull-scalp interfaces, which resulted in only a partial correction of the skull-induced aberrations. The memory effect of skull-induced distortions in PA signals arising within a small “isoplanatic” intracranial region was exploited to de-aberrate images of a collection of point-absorbing targets through a piece of excised skull obtained from the fronto-temporal region^26^. However, it relied on the invasive prior measurement of a point source signal through the skull. Several other approaches have been proposed in the PACT literature, but they have only been demonstrated with a simian skull^20,21^ or an acrylic globe^22,23^, which induce significantly less distortion than an adult human skull. Skull de-aberration is also an active area of research in the transcranial focused ultrasound (tFUS) community^29–34^. However, in tFUS, the focus is typically away from the skull, which results in near-normal incidence and minimal shear conversion, thus allowing for acoustic-only models. In contrast, in PACT, the acoustic sources are close to the skull and span a large field-of-view, thus necessitating elastic wave models with high accuracy over a large domain. Finally, several simulation studies have also been conducted to study the problem of skull-induced acoustic aberrations^25,35,36^.

Here, we demonstrate the de-aberration of PACT images of light-absorbing phantoms of vasculature through an ex-vivo adult human skull using a homogeneous elastic model for the skull. Using only the geometry of the skull, obtained from adjunct imaging data (X-ray computed tomography or magnetic resonance imaging), along with its position and orientation, we can achieve high-fidelity aberration correction in the phantom images, in terms of the recovered features. Our work addresses the longstanding problem of skull aberration in transcranial PACT and takes us one step closer to achieving the goal of human brain PACT.

## Results

We imaged light-absorbing phantoms through an ex-vivo adult human skull using a three-dimensional (3D) PACT system similar to the one described here^37^. We placed the phantoms close to the inner surface of the ex-vivo skull to mimic cortical blood vessels. We took special care to remove any trapped air within the skull trabeculae (see Methods). We use two illumination approaches—internal and external illumination, respectively. The internal illumination leads to an improved signal-to-noise ratio (SNR) in the PA signals, thus allowing us to effectively test our de-aberration method, whereas the external illumination is used to demonstrate our method in noninvasive transcranial PACT (light is delivered from outside the skull, mimicking the clinical transcranial setting, as opposed to the internal illumination where light is delivered directly to the target). The external illumination is also used to acquire a PA image of the fiducial markers on the skull, which is used to co-register the X-ray computed tomography (CT) image of the skull with the PACT frame of reference.

**Fig. 1:**
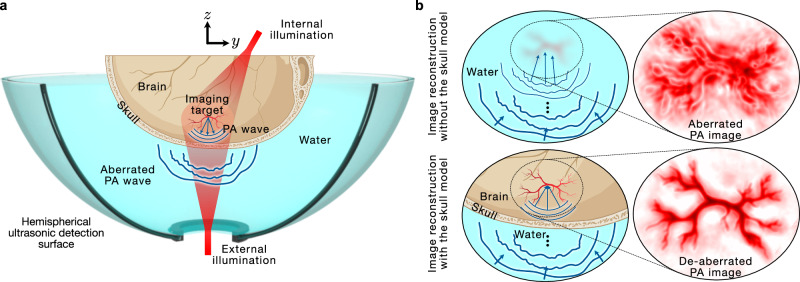
Schematic of the experimental setup and the image reconstruction scheme. **a** 3D PACT system comprising a hemispherical ultrasonic detection surface (obtained by rotating four arc-shaped ultrasonic transducer arrays) filled with water. The ex-vivo skull is placed within the imaging field of view. Internal illumination is used to improve the SNR in this experiment, which allows us to effectively test our de-aberration method. External illumination is used to acquire an image of the skull fiducial markers for co-registration and to demonstrate our method in noninvasive transcranial PACT. The PA wave generated from the imaging target gets aberrated by the skull before reaching the ultrasonic detection surface. **b** Illustration of image reconstruction with and without skull modeling. (Top) Reconstructing an image of the target transcranially without accounting for the skull leads to an aberrated PA image. (Bottom) Incorporating the effect of the skull in the reconstruction method leads to the correction of the aberrated wavefronts, thus resulting in a de-aberrated PA image.

A schematic of the experimental setup is shown in Fig. 1a, which illustrates the skull, the imaging target, the two illumination approaches, and the hemispherical ultrasonic detection surface, which was obtained by rotating four arc-shaped ultrasonic transducer arrays. PA waves originate from the imaging target and are aberrated by the skull before reaching the ultrasonic detectors. Reconstructing an image without accounting for the skull leads to an aberrated PA image, as shown in the top row in Fig. 1b. On the other hand, an image reconstruction method that incorporates the skull model can effectively refocus the aberrated waves, thus resulting in a de-aberrated PA image.

**Fig. 2:**
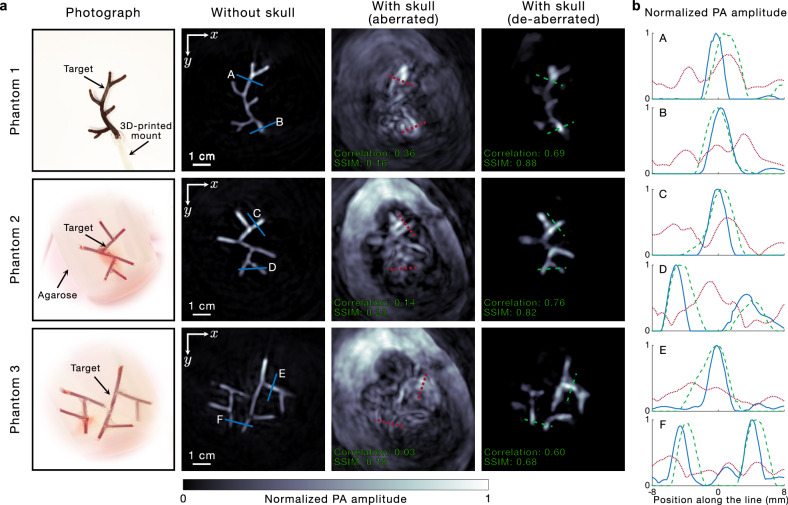
Experimental demonstration of skull de-aberration in PACT. **a** Images of three light-absorbing phantoms are acquired with and without the presence of the ex-vivo skull using internal illumination. The photographs of the phantoms are shown in the left-most column as the ground truth. The phantoms are 1–2 mm thick and within a 5 cm × 5 cm area. The UBP images of the phantoms acquired in the absence and presence of the skull are shown in the second and third columns, respectively. Finally, the de-aberrated images acquired through the skull are shown in the right-most column. The de-aberrated images remarkably recover most of the features that are seen in the respective ground truth (i.e., the photographs and the UBP images acquired without the skull), whereas the UBP images acquired in the presence of the skull hardly reveal any of the features. The PACT images are presented as MAPs of the relevant slices in the respective reconstructed volume along the $z$ direction. The correlations and SSIMs of the aberrated and de-aberrated phantom images with their respective skull-less images are shown in the bottom-left of each panel. **b** Plots of the line profiles extracted from the images of the phantoms (profiles [A, B] for phantom 1, [C, D] for phantom 2, and [E, F] for phantom 3). The colors and line styles of the respective profiles match the plotted lines in (**a**).

We reconstruct the images of several phantoms acquired in the absence and presence of the skull using UBP, and acquired in the presence of the skull using our approach and present them along with their respective ground truth (i.e., their photographs) in Fig. 2a. The images are self-normalized by their respective maximum voxel value and presented as maximum amplitude projections (MAPs) of the relevant slices in the respective reconstructed volume along the $z$ direction. Phantom 1 is made of polylactic acid (PLA), whereas phantoms 2 and 3 are made of blood-filled tubes (1.68 mm inner diameter) embedded in agarose. The phantoms used in this paper are designed to be 1–2 mm thick and within an area of 5 cm $\times$ 5 cm. We see in the case of all three phantoms that the images acquired in the presence of the skull, reconstructed using UBP (which does not account for the impact of the skull), are severely aberrated and hardly capture any of the features of the respective targets. Remarkably, for the same phantoms, the de-aberrated transcranial images, shown in the right-most column in Fig. 2a, recover nearly all the features that can be seen in the respective ground truth images as well as the UBP images of the phantoms acquired in the absence of the skull. To quantify the improvement due to the aberration correction, we computed the correlation coefficients and structural similarity index measures (SSIMs) of the aberrated and de-aberrated phantom images with their corresponding skull-less images. The correlations of the three phantoms in Fig. 2 improved from 0.36, 0.14, and 0.03, respectively, for the aberrated images to 0.69, 0.76, and 0.60, respectively, for the de-aberrated images. Similarly, the SSIMs improved from 0.16, 0.23, and 0.18, respectively, for the aberrated images to 0.88, 0.82, and 0.68, respectively, for the de-aberrated ones. Further, we extract two line profiles from the images of each phantom and plot them in Fig. 2b, which shows that the de-aberrated phantom images closely resemble the images acquired in the absence of the skull. A detailed description of the preprocessing and image reconstruction scheme is presented in Methods.

**Fig. 3:**
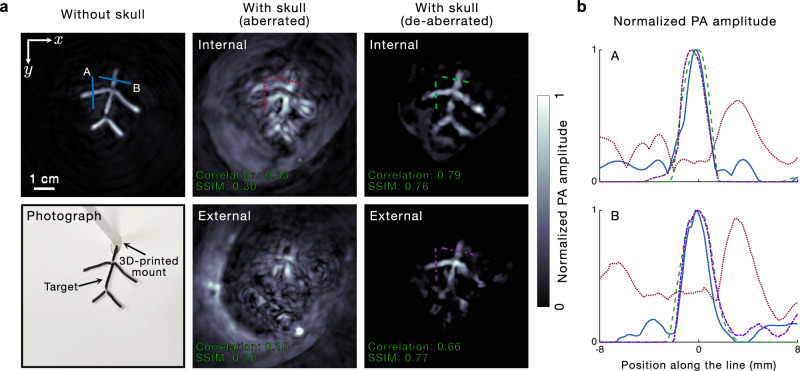
De-aberration in noninvasive imaging through an ex-vivo adult human skull. **a** UBP image in the absence of the skull, a photograph, UBP (aberrated) images in the presence of the skull obtained with internal and external illumination, and the de-aberrated images in the presence of the skull with internal and external illumination, respectively. The PACT images are presented as MAPs of the relevant slices in the respective reconstructed volume along the $z$ direction. The correlations and SSIMs of the aberrated and de-aberrated phantom images with their respective skull-less images are shown in the bottom-left of each panel. **b** Line profiles (A and B, as shown in the top-left panel of (**a**)) extracted from the images in (**a**).

The images in Fig. 2 were obtained by internally illuminating the targets to ensure high SNR. However, we can also de-aberrate noninvasively through the skull using external illumination, as shown in Fig. 3. In Fig. 3a, we present the UBP image of a phantom (made of black wires of ~1.5 mm thickness) in the absence of the skull, along with its photograph. Then, we show the aberrated and de-aberrated images obtained using internal and external illuminations, respectively, thus demonstrating the noninvasive de-aberration of the phantom image through the skull. The correlations of the aberrated and de-aberrated images obtained using internal illumination with the skull-less image are 0.33 and 0.79, respectively, whereas those using external illumination are 0.10 and 0.66, respectively. Similarly, the SSIMs of the aberrated and de-aberrated images obtained using internal illumination with the skull-less image are 0.30 and 0.76, respectively, whereas those using external illumination are 0.20 and 0.77, respectively. We also extract line profiles from the phantom images in Fig. 3b, which show that the de-aberrated images obtained using external and internal illuminations, respectively, are comparable to the image obtained in the absence of the skull, in terms of the resolution and recovered phantom position. An interesting observation from Fig. 3a is that while the phantom is visible, albeit distorted, in the aberrated image obtained using internal illumination, it is hardly discernible from the background in the aberrated image obtained using external illumination. This is because of the background generated by the skull, which is much stronger in the external illumination case than in the internal illumination one. This deterministic interference from the skull is hugely detrimental to ex-vivo transcranial PACT—much more than random noise, which can be mitigated by averaging—since it dominates the weaker signals arising from within the skull. In the in-vivo case, a similar deterministic background is generated by the strong signal generated in the scalp and its reflection from the skull. However, as we have shown, we can partially overcome the background by correcting the skull-induced aberrations. This correction refocuses the target signals, significantly enhancing their amplitude relative to the background clutter, thereby making the target features discernible. To further validate our approach, we imaged a blood-tube phantom using external illumination (results presented in Supplementary Fig. 1). However, due to the strong interference signal from the skull, only select segments of the phantom were successfully recovered.

**Fig. 4:**
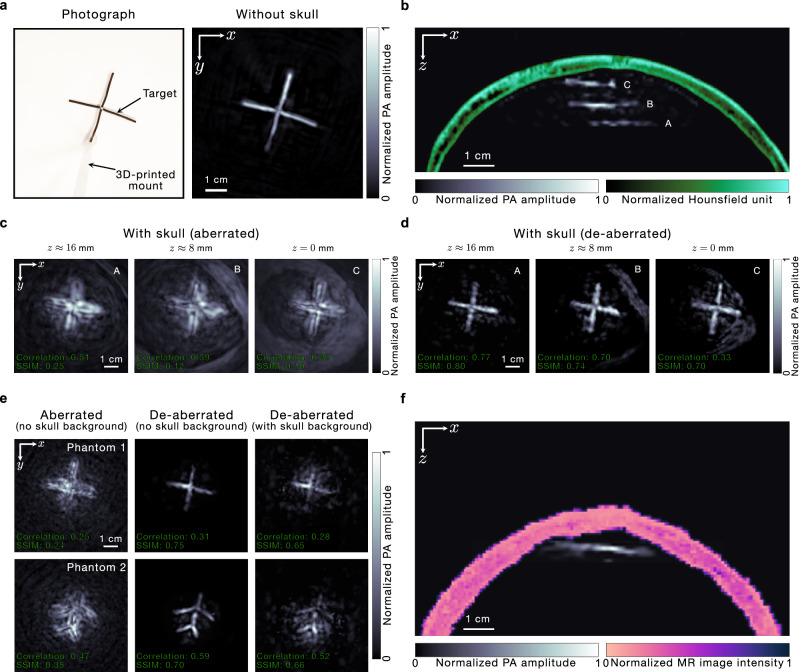
De-aberration at different phantom positions relative to the skull and for a second ex-vivo skull. **a** Photograph and a UBP image in the absence of the skull, respectively, of a phantom. **b** Cross-sections of the de-aberrated PACT images of the phantom acquired at different positions relative to the skull overlaid on a cross-section of the registered X-ray CT image of the ex-vivo skull. Phantom position C is closest to the skull and is set as the $z=0$ plane. **c**, **d** Aberrated (**c**) and de-aberrated (**d**) images (MAPs) of the phantom, respectively, acquired in the presence of the skull, at different $z$ positions (A–C, as shown in (**b**)) relative to the skull. The aberrated images in (**c**) are reconstructed using UBP. **e** Left and middle columns: aberrated and de-aberrated images (MAPs) of two phantoms without the skull background, respectively, acquired in the presence of the second ex-vivo skull. Right column: de-aberrated images (MAPs) of the two phantoms with the skull background, acquired in the presence of the second ex-vivo skull. **f** Cross-section of the de-aberrated image of phantom 1 in (**e**) (without the skull background) overlaid on the cross-section of the MR image of the second ex-vivo skull.

We also acquire the images of a phantom (made of black wires of ~1.5 mm thickness) through the skull at different positions along the $z$ direction using internal illumination. This is done in order to test our de-aberration approach for signals arising from different positions within the skull. The photograph and the UBP image in the absence of the skull, respectively, of the phantom are shown in Fig. 4a. In Fig. 4b, we show the cross-sections (in the $x$–$z$ plane) of the de-aberrated PACT images of the phantoms acquired at the three positions (within 16 mm along the $z$ direction) overlaid on a cross-section of the registered X-ray CT image of the ex-vivo skull. This shows the positions of the phantoms relative to the ex-vivo skull. Further, as evidenced in Fig. 4b, the average thickness of the relevant portion of the ex-vivo skull is roughly 6.5 mm. At each position, we reconstruct the aberrated (UBP) and de-aberrated images and present them in Fig. 4c, d, respectively. These images demonstrate that the de-aberration is effective for different depths relative to the skull. This consistency is also seen in the correlation coefficients and SSIMs for the aberrated and de-aberrated images with respect to the skull-less images, which are provided in the bottom-left of the respective images. We have also prepared Supplementary Fig. 2, which shows the MAPs of the reconstructed volumes of the phantom at different depths from different viewing angles in 3D.

To further demonstrate the fidelity of our de-aberration approach, we acquire images of phantoms through another ex-vivo skull (using external illumination). We extracted the shape of the second ex-vivo skull using magnetic resonance (MR) imaging (see Methods), instead of X-ray CT (which was used for the first ex-vivo skull), primarily to demonstrate that an imaging modality that does not use ionizing radiation could also be used to obtain the skull geometry. Since our objective is to demonstrate the de-aberration of the phantom images, we isolate the transcranial phantom signal by subtracting a skull-only signal from the total acquired signal, which reduces the interference from the skull background, akin to the internal illumination images. We present the aberrated (UBP) and de-aberrated images of two phantoms obtained without the skull background in the first two columns of Fig. 4e. These images show that we can de-aberrate phantom images reliably through either of the ex-vivo skulls and demonstrate the robustness of this approach. We also show the de-aberrated images of the two phantoms obtained with the skull background in the third column, which, like Fig. 3b, exhibit more background than those without the skull background but still reveal most of the respective phantom features accurately. A cross-section (in the $x$-$z$ plane) of the de-aberrated image (without the skull background) of phantom 1 in Fig. 4e overlaid on the cross-section of the MR image of the second ex-vivo skull is shown in Fig. 4f. The stark improvement in the de-aberrated images across different levels of phantom complexity, different phantom positions, and even different skulls—which exhibit natural variations in shape, structure, and properties—shows the reliability and generality of our reconstruction scheme. The correlation coefficients and SSIMs of the aberrated and de-aberrated images with respect to the skull-less images are shown in the bottom-left of the respective images.

**Fig. 5:**
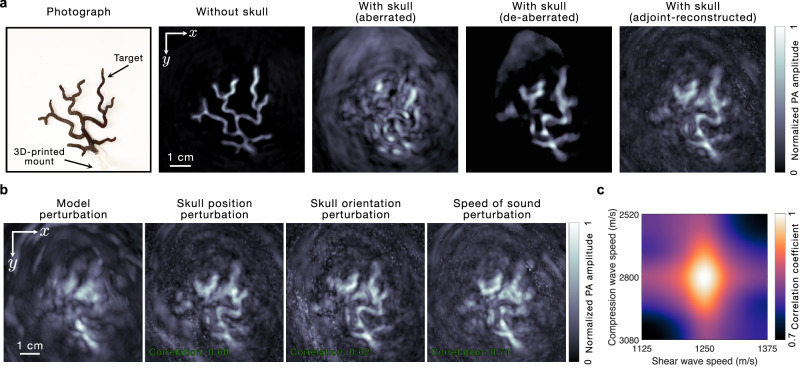
Impact of model-mismatch on de-aberration. **a** Photograph of a phantom along with its UBP images (MAPs) in the absence and presence of the skull, respectively, and its de-aberrated and adjoint-reconstructed images (MAPs) in the presence of the skull, respectively. **b** Adjoint-reconstructed images (MAPs) under different conditions of model-mismatch. The leftmost image (model perturbation) is reconstructed by modeling the skull as a medium that only supports compression waves (i.e., an acoustic model), and it shows the importance of modeling the shear component. The second, third, and fourth images are reconstructed using the elastic model by perturbing the skull position (CC: 0.60 at a perturbation of (1, −1, 0) cm), orientation (CC: 0.62 at a perturbation of 10° about the $y$-axis), and compression and shear speeds (CC: 0.71 at compression and shear speeds of 2520 m s^−1^ and 1375 m s^−1^, respectively). These three images do not show a severe degradation of image quality and demonstrate that our method is robust to errors in these parameters. **c** Plot of the correlation coefficients (with respect to the adjoint-reconstructed image in (**a**)) of the images reconstructed with different compression and shear speeds within the skull. It shows that changes in the shear speed seemingly affect the image quality more than the changes in the compression speed.

The performance of any model-based image reconstruction approach depends on the accuracy of the underlying model. The accuracy of our model depends on the correctness of the position, orientation, and properties of the skull. To test the sensitivity of our method to errors in these properties, we perturb each of these quantities and study their effect on the de-aberration in Fig. 5. In Fig. 5a, we show the photograph of a phantom (made of PLA) along with its UBP images (MAPs) in the absence and presence of the skull, respectively, and its de-aberrated (using the iterative reconstruction method described in Methods) and adjoint-reconstructed (see Methods) images (MAPs) in the presence of the skull, respectively. For evaluating the effect of model-mismatch, we consider the adjoint-reconstructed image (and present them as MAPs) under different scenarios. We use the adjoint-reconstructed image for this analysis because it is a linear operation that does not depend on regularization parameters. This allows for a direct comparison of the model’s focusing capability under different perturbations without the confounding effects or potential bias introduced by the non-linear regularizers used in the full iterative method. First, we evaluate the effect of ignoring shear waves by modeling the skull as a medium that only supports compression waves (i.e., an acoustic model) and present this image in Fig. 5b as a model perturbation. The noticeable deterioration in the reconstructed image quality shows the importance of modeling the shear component. To study the impact of errors in the skull position in the elastic model on de-aberration, we translate the skull by a maximum of around 1 cm in different directions and pick the image that has the least correlation coefficient (CC) with the image obtained without translation. We present this image (CC: 0.60 at a perturbation of (1, −1, 0) cm) in Fig. 5b. Since a shift in the image position negatively affects the CC, we consider a “sliding correlation”, which is computed as the maximum of the normalized cross-correlation between the two images. We perturb the orientation of the skull by rotating it about the coordinate axes by ±10°. Once again, we pick the image with the least correlation with the image obtained without rotation and present it (CC: 0.62 at a perturbation of 10° about the $y$-axis) in Fig. 5b. We also change the assigned compression and shear speed in the skull by ±10% and present the image with the least correlation (CC: 0.71 at compression and shear speeds of 2520 m s^−1^ and 1375 m s^−1^, respectively) in Fig. 5b. Notably, although the skull position, orientation, and speed of sound perturbation result in a degradation in the image quality, many of the features of the phantom are still visible. This shows that while the accuracy of the skull parameters is necessary for optimal de-aberration, small errors in these parameters may still result in comparable images. Finally, we plot the correlation coefficients (with respect to the adjoint-reconstructed image in Fig. 5a) for different assigned values of compression and shear speeds within the skull in Fig. 5c. While errors in the speeds of both polarizations degrade image quality, the reduction in the correlation coefficient is noticeably faster with the shear speed compared to proportional changes in the compression speed, thus showing that changes in the shear speed seem to affect the image quality more than changes in the compression speed. Since the correlation coefficient plots for the position and orientation perturbation were not elucidative, and they depend on the phantom placement relative to the skull, making them less generalizable, we have not included them here.

## Discussion

Here, we presented an experimental demonstration of high-fidelity de-aberration of PACT images of blood-filled phantoms through an ex-vivo skull, which addresses a long-standing challenge faced in PACT. Using a homogeneous elastic model for the skull, we achieved successful de-aberration, in terms of feature recovery, for phantoms with different levels of complexity. We demonstrated the same degree of de-aberration noninvasively, i.e., by illuminating the phantoms from outside the skull. The presented approach can be utilized for transcranial imaging at different depths, as shown by the results for different phantom positions relative to the skull. We have also demonstrated the generality of our method by showing a similar extent of aberration correction through a second ex-vivo adult human skull. Finally, to ensure the usability of the proposed method in a practical setting, we investigated the sensitivity of our de-aberration approach to various model parameters. While modeling the shear component was found to be crucial to de-aberration, the model was resilient to small errors in the skull position, orientation, and speeds, with the shear speed affecting the performance more than the compression speed. Our method only requires the geometry, position, and orientation of the skull. For the ex-vivo phantom experiments, the geometry of the skull was obtained using X-ray CT for the first skull and MR imaging for the second skull, whereas the position and orientation of the skull in the PA frame of reference were estimated using fiducial markers on the surface of the skull. The compression and shear speeds of the skull were tuned within their respective physiological ranges. These results convincingly demonstrate the correction of skull-induced aberrations in transcranial PACT, thus establishing its potential as a complementary high-resolution neuroimaging modality to fMRI.

Our approach holds several technical and practical advantages over existing methods in the literature, such as the layered back-projection^24^ method (LUBP) and the memory effect-based method^26^. Unlike LUBP, which ignores the polarization of shear waves, reflections from and within the skull, and uses multiple approximations to handle the wave propagation, our approach follows a full-wave modeling scheme, which handles the fluid-solid interface more accurately, is adaptable to inhomogeneous models of the skull, and can be used in an iterative framework that can compensate for modeling errors by leveraging prior information. These advantages result in a much improved de-aberrated image quality from our method compared to LUBP, as evidenced in Supplementary Fig. 3. The memory effect-based approach, on the other hand, requires an invasive calibration measurement and is valid only within a small region, thus necessitating several invasive calibration measurements for imaging a large region. In contrast, our approach, which only requires the geometry, position, and orientation of the skull (obtainable via standard X-ray CT or MR imaging), along with representative values for the skull properties, is much more clinically viable. Finally, although the isotropic elastic wave equation has been proposed before to model the skull^22,23^, our work demonstrates its first application for successful aberration correction through an adult human skull in transcranial PACT.

The geometries of the two ex-vivo skulls used in this work were obtained using X-ray CT (for the first skull) and MR imaging (for the second skull), respectively, and fiducial markers on each skull were used to register the skull in the PACT frame of reference. Even in the in-vivo case, X-ray CT can be used to obtain the shape of the skull. Alternatively, certain MR imaging sequences may be used to directly obtain the skull geometry^38–40^. To register the skull in the PACT frame of reference, an additional MR angiography scan of the head can be performed. The PACT frame of reference can be registered with the MR frame of reference using a previously established framework^15^, which uses superficial scalp arteries that appear in both the PACT and MR angiography images of the head. The MR frame of reference can then be registered with the X-ray CT image using multimodal medical image registration algorithms^41^. Alternatively, photogrammetry^42^ may be used to register the PACT and MR frames of reference.

PACT of the human brain, coupled with our de-aberration approach, has attractive clinical potential. While the optical scattering of brain tissue limits the penetration depth compared to the water-filled skull used in this study, previous studies^13,15^ have concluded that adequate SNR is achievable in in-vivo transcranial PACT with sufficient illumination (within the safety limit) and well-designed ultrasound detector arrays. In the current work, PACT may be a complementary technique to X-ray CT or MR imaging since one of these modalities is first used to estimate the skull parameters. Nonetheless, PACT can be subsequently used to monitor brain function or other clinically relevant information and to aid in the diagnosis and management of brain diseases such as traumatic injuries, tumors, and strokes^43–45^. This is particularly beneficial since PACT does not involve exposure to harmful ionizing radiation or administration of contrast agents. PACT is well-suited for patients with either fMRI-incompatible implants or claustrophobia, or patients who need frequent bedside monitoring at a lower cost. In the future, a combined PA-ultrasound tomography (UST) imaging system is envisioned in which ultrasound imaging is used to image the properties and shape of the skull. Such a system would allow for the in-situ estimation of the skull’s acoustic properties and geometry within the same coordinate frame, thereby automating the parameter estimation process and eliminating the need for external X-ray, CT or MR imaging scans and the associated co-registration steps. This would significantly reduce the effort and resources required, lowering the barriers for wider clinical adoption.

A few aspects can be further improved. Firstly, we can use a heterogeneous model for the skull. Although our approach is readily extendable to the heterogeneous case, it is challenging to estimate the elastic properties of the skull accurately. While some literature exists on mapping the Hounsfield units in X-ray CT images to the compression and shear wave speeds^46–49^, a consensus on the optimal solution still does not exist^50^, especially for the shear wave speed. Estimating the skull properties from an MR image is another important direction since it obviates the need for an X-ray CT image, which involves ionizing radiation. Second, we did not consider the effects of dispersion and frequency-dependent attenuation within the skull in our model^13^. Incorporating these effects is a promising future direction and is expected to improve the accuracy of the skull model, potentially resulting in better de-aberration and a higher resolution. Finally, we need to study the effect of the PA signals arising outside the skull (e.g., hair follicles and melanin in the human scalp), which get reflected from the skull and potentially corrupt the cortical signals. Accounting for these reflections is the next major challenge in in vivo transcranial PACT and will be the focus of our future work.

## Methods

### Sample preparation

The two ex-vivo human skulls (Skull Unlimited International Inc.) used in this study were donated by an 83-year-old male and a 51-year-old female, respectively. We immersed each ex-vivo skull in water for more than 2 h before the experiment, and the skull cavity was filled with water during imaging. We used a vacuum pump to remove any trapped air in the skull trabeculae. The MR image of the second ex-vivo skull, submerged in deionized water to create a negative contrast, was acquired using a 3 T Siemens Prisma. Fit scanner with a 32-channel head receive coil at the Caltech Brain Imaging Center. A multi-echo rapid gradient echo (MERAGE) sequence was used with a root mean square (RMS) echo combination (repetition time = 2210 ms, RAGE echo time = 1.6, 3.5, 5.3, and 7.1 ms, flip angle = 8°, in-plane generalized autocalibrating partially parallel acquisitions (GRAPPA) acceleration factor = 2, pixel bandwidth = 700 Hz, 0.9 × 0.9 × 0.9 mm^3^ isotropic voxel, 3D distortion correction, total image acquisition time = 247 s). The phantoms used in this paper were either made of black wires (~1.5 mm thickness), were 3D-printed using polylactic acid, or were made of blood-filled tubes embedded in agarose. The phantoms used in this paper were designed to be 1–2 mm thick and within an area of 5 cm × 5 cm. The blood tube phantoms consisted of a micro-renathane tubular structure (1.68 mm inner diameter, Braintree Scientific) filled with bovine blood (B-C8080, defibrinated, QuadFive) that was sealed from all ends using hot melt adhesive and embedded in 2.5% agarose (A9414, low gelling temperature, MilliporeSigma). While the blood-filled tube phantoms accurately mimic the optical properties in an in-vivo setting, the other materials allowed us to conveniently test our method with stable (long shelf-life) and highly complicated structures. Custom-designed 3D-printed mounts were used to ensure the repeatability of the phantom position. The PACT system^37^ consists of four arc-shaped ultrasonic transducer arrays (Imasonic, Inc.) with a central frequency of 2.25 MHz and a one-way bandwidth of >90%. The reconstruction algorithm uses 4 arcs with 128 elements, creating a hemispherical detection view with a radius of 13 cm. For image acquisition, the arrays were rotated 90 degrees in 100 steps over 5 s. At each step, the photoacoustic signals were measured once (no averaging). The raw data was sampled at 10 MHz with a length of 4096 samples. For PA wave excitation, 1064 nm light below the ANSI safety limit was diffused onto the phantoms using external (LPY7875, Litron; maximum pulse energy: ~2.5 J) and internal (Q-Smart 850, Quantel; maximum pulse energy: ~0.8 J) illuminations, respectively.

### Data processing and image reconstruction

The acquired PA signals were low-pass filtered^51^ using a second-order Butterworth filter with a cutoff frequency of 0.5 MHz. Beyond this frequency, only a minor image quality improvement was observed, likely due to the dispersion, frequency-dependent attenuation, and heterogeneity of the skull. The Kabsch algorithm^52^ was used to estimate a rigid transformation between the X-ray CT (or MR) image of the skull and the PA frame of reference. The transformed CT (or MR) image was then binarized, and the holes within the binarized skull were filled in using morphological operations^53^. It was then treated as a homogeneous elastic medium. The background medium was water. The compression and shear wave speeds within each individual skull were tuned within their respective physiological ranges^13^ to optimize the image quality. The sound speed in water was estimated from its temperature measurement^54^.

We used the isotropic elastic wave equation to model wave propagation in the skull, as given by the following initial value problem^55^ (written in index notation^56^):

1 $$\rho {\partial }_{t}{v}_{i} = 	\, {\partial }_{j}{\sigma }_{{ij}},\\ {\partial }_{t}{\sigma }_{{ij}} = \,	\lambda {\delta }_{{ij}}{\partial }_{k}{v}_{k}+\mu \left({\partial }_{i}{v}_{j}+{\partial }_{j}{v}_{i}\right),\,{\mbox{and}}\\ {\sigma }_{{ij}}{|}_{t=0} = 	-{p}_{0}{\delta }_{{ij}},{v}_{i}{|}_{t=0}=0,$$

where ${\sigma }_{{ij}}$ is the induced stress tensor at time $t\ge 0$, ${v}_{i}$ is the velocity vector ${\partial }_{i}\equiv \partial /\partial {x}_{i}$ (${x}_{i}$ denotes the ${i}^{{\mbox{th}}}$ Cartesian position coordinate for $i\in \{{{\mathrm{1,2}}},\,3\}$), ${\partial }_{t}$ denotes a temporal derivative, ${\delta }_{{ij}}$ is the Kronecker delta, ${p}_{0}$ is the initial pressure, $\rho$ is the mass density, and $\lambda$ and $\mu$ are the Lamé parameters.

The images are reconstructed by solving the following regularized least squares problem,

2 $${\hat{p}}_{0}={{{{\rm{argmin}}}}}_{{p}_{0}\in {{\mathbb{R}}}_{\ge 0}^{N}}\frac{1}{2}{{||}{SA}{p}_{0}-d{||}}^{2}+R\left({p}_{0}\right),$$

where $S:{{\mathbb{R}}}^{{N}_{x}\times {N}_{t}}\to {{\mathbb{R}}}^{{N}_{m}\times {N}_{t}}$ is a sampling operator that maps the pressure at ${N}_{x}$ positions and ${N}_{t}$ time instances to the ${N}_{m}$ ultrasonic transducers’ measurements, $A:{{\mathbb{R}}}^{N}\to {{\mathbb{R}}}^{{N}_{x}\times {N}_{t}}$ is a discrete approximation for the operator that solves the initial value problem in Eq. 1, $N$ is the number of 3D positions at which the initial pressure is being solved for, ${\hat{p}}_{0}$ is the estimated initial pressure distribution, ${{\mathbb{R}}}_{\ge 0}^{N}$ is the set of $N$-dimensional real vectors with non-negative entries, $d\in {{\mathbb{R}}}^{{N}_{m}\times {N}_{t}}$ is the data measured by the transducers, and $R:{{\mathbb{R}}}^{N}{\mathbb{\to }}{\mathbb{R}}$ is the regularizing function, chosen here to be a linear combination of the ${L}_{1}$-norm and the isotropic total variation (TV) semi-norm^57^. We solve the above optimization problem using an accelerated proximal gradient method^57–59^, where the step size can be estimated using power iterations^60^. The adjoint-reconstructed images in Fig. 4 are reconstructed by computing the action of the adjoint of ${SA}$ on $d$, which is equivalent to performing a single iteration in the absence of any regularizers. At the gradient step, the actions of the operator ${SA}$ and its adjoint, respectively, are evaluated using the pseudo-spectral time-domain-based k-Wave toolbox^61^. The spatial discretization was 0.5 mm, which is roughly one-sixth of the wavelength of sound in water at the maximum frequency of consideration. The temporal step size was 50 ns, and the computational grid was terminated with a perfectly matched layer to avoid spurious reflections from the edges of the domain. The density, compressional wave speed, and shear wave speed of the first ex-vivo skull were set to $1850\,{{\mbox{kg}}\,{\mbox{m}}}^{-3}$, $2800\,{\mbox{m}}{{\mbox{s}}}^{-1}$, and $1250\,{\mbox{m}}{{\mbox{s}}}^{-1}$, respectively, and of the second ex-vivo skull were set to $1850\,{{\mbox{kg m}}}^{-3}$, $2800\,{\mbox{m}}{{\mbox{s}}}^{-1}$, and $1400\,{\mbox{m}}{{\mbox{s}}}^{-1}$, respectively. The parameter tuning was performed using a non-iterative adjoint reconstruction approach instead of the iterative algorithm to ease the computational burden. It is worth noting that because we employ a homogeneous model for the heterogeneous skull, the optimized acoustic speeds represent effective values. These values depend on the specific structural composition (e.g., the ratio of cortical to trabecular bone thickness) of the individual skull, which explains the variation in optimal speeds observed between the two skulls used in this study. The weights of the two regularizers were tuned for the different illumination conditions (internal and external illuminations), different imaging target types (black wires, PLA, and blood tubes), and different skulls. This is necessary since the regularizers are sensitive to various factors such as the signal-to-interference ratio and overall signal scale, which vary under these different conditions. The images were reconstructed within a volume of 80 × 80 × 20 mm³, with an isotropic voxel size of 0.5 mm. The optimization terminated after 10 iterations, with an average run time of 34 min and 30 s per iteration, when using a single GPU. A detailed reconstruction algorithm is provided in Supplementary Methods 1. The data processing and image reconstruction were performed using MATLAB R2023b on an Ubuntu 24.04.1 LTS system equipped with dual Intel Xeon 6248 R CPUs (24 cores per CPU, 3.0 GHz base, 4.0 GHz Turbo), 12 × 128 GB DDR4-2933 ECC memory, and dual NVIDIA A100 GPUs (PCI-E 4.0, 40GB HBM2 memory each).

### Reporting summary

Further information on research design is available in the Nature Portfolio Reporting Summary linked to this article.

## Supplementary information

Supporting Information

Reporting Summary

## Data Availability

The data that support the findings of this study are provided within the paper and its Supplementary materials. To enable reproduction of the results, the raw photoacoustic data for a representative phantom (Fig. 3, acquired with both internal and external illumination) and the corresponding X-ray CT data of the skull have been deposited in Zenodo (10.5281/zenodo.17931887).

## Code availability

A detailed step-by-step description of the reconstruction algorithm and the specific reconstruction settings are provided in the paper and its Supplementary materials. The reconstruction code is not publicly available because it is proprietary and may be used in licensed technologies.
