# Supplementary material for: De-aberration for noninvasive transcranial photoacoustic computed tomography through an adult human skull
Source: Commun Phys. 2026 Feb 14;9(1):116. doi: 10.1038/s42005-026-02545-3 (PMC13043303; doi:10.1038/s42005-026-02545-3)
Supplement: Supplementary file 1 — Supporting Information [file 42005_2026_2545_MOESM1_ESM.pdf]

Supplementary Information

De-aberration for noninvasive transcranial photoacoustic computed tomography through an adult human skull

Yousuf Aborahama[1,†], Karteekeya Sastry[1,2,†], Manxiu Cui[1,2,†], Yang Zhang[1], Yilin Luo[1,2], Rui Cao[1], Geng Ku[1], Jigmi Basumatary[1], Junhao Zhu[1,2], Siying Kong[1,2], Lihong V. Wang[1,2,*]

[1]Caltech Optical Imaging Laboratory, Andrew and Peggy Cherng Department of Medical Engineering, California Institute of Technology, 1200 East California Boulevard, Pasadena, CA 91125, USA.

[2]Caltech Optical Imaging Laboratory, Department of Electrical Engineering, California Institute of Technology, 1200 East California Boulevard, Pasadena, CA 91125, USA.

[†]These authors contributed equally to this work.

[*]Correspondence: LVW@caltech.edu

Contents

Supplementary Fig. 1 | De-aberration in noninvasive imaging of a blood-tube phantom through an ex-vivo adult human skull.

Supplementary Fig. 2 | MAPs of the reconstructed phantom images in Figs. 4c and 4d along different directions to show the 3D structure.

Supplementary Fig. 3 | Comparison of LUBP (1) with our de-aberration method.

Supplementary Methods 1 | Reconstruction algorithm.

Supplementary Figures

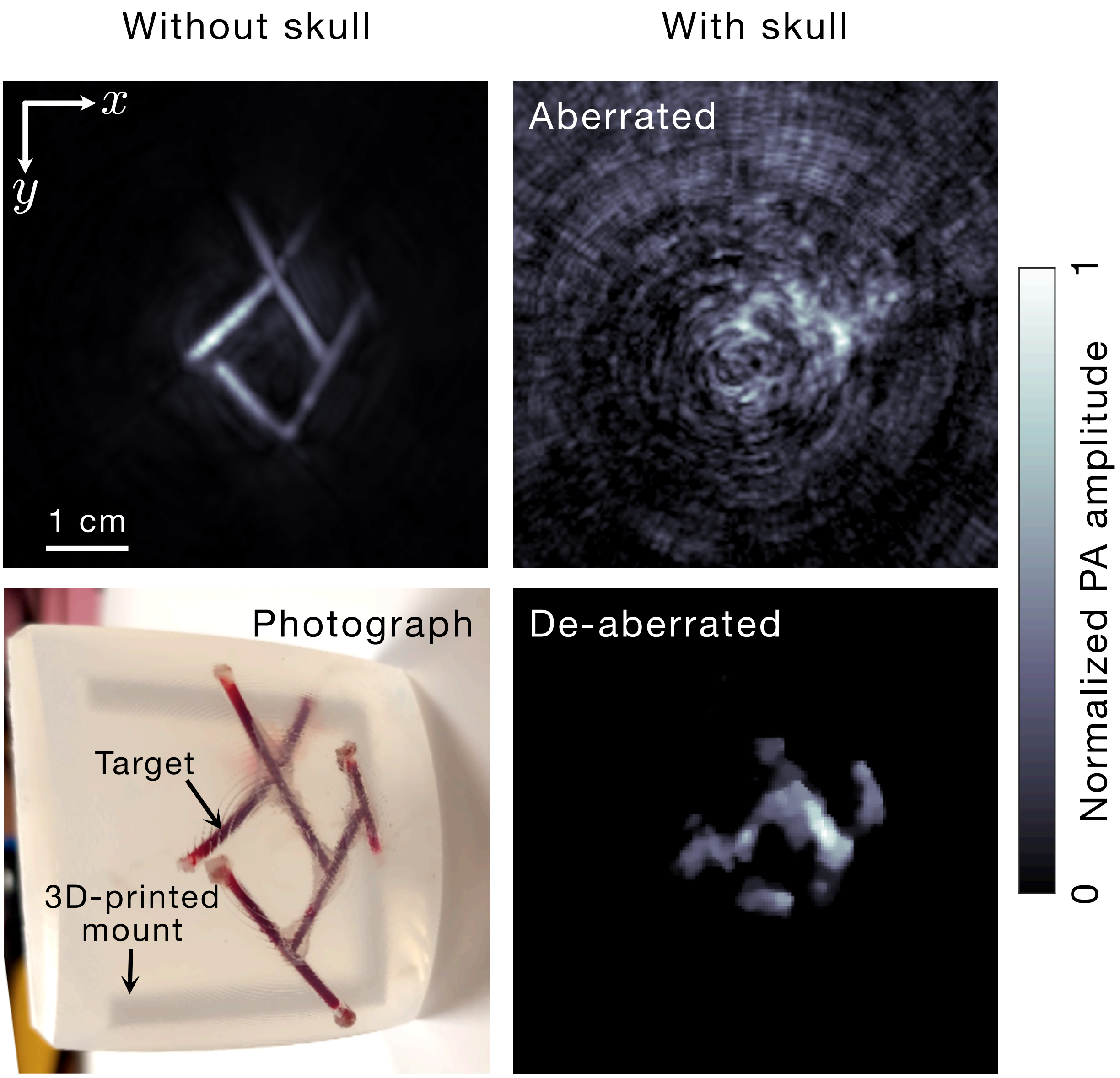

Supplementary Fig. 1 | De-aberration in noninvasive imaging of a blood-tube phantom through an ex-vivo adult human skull. UBP image in the absence of the skull, photograph, UBP (aberrated) image in the presence of the skull obtained with external illumination, and the de-aberrated image in the presence of the skull with external illumination, respectively.

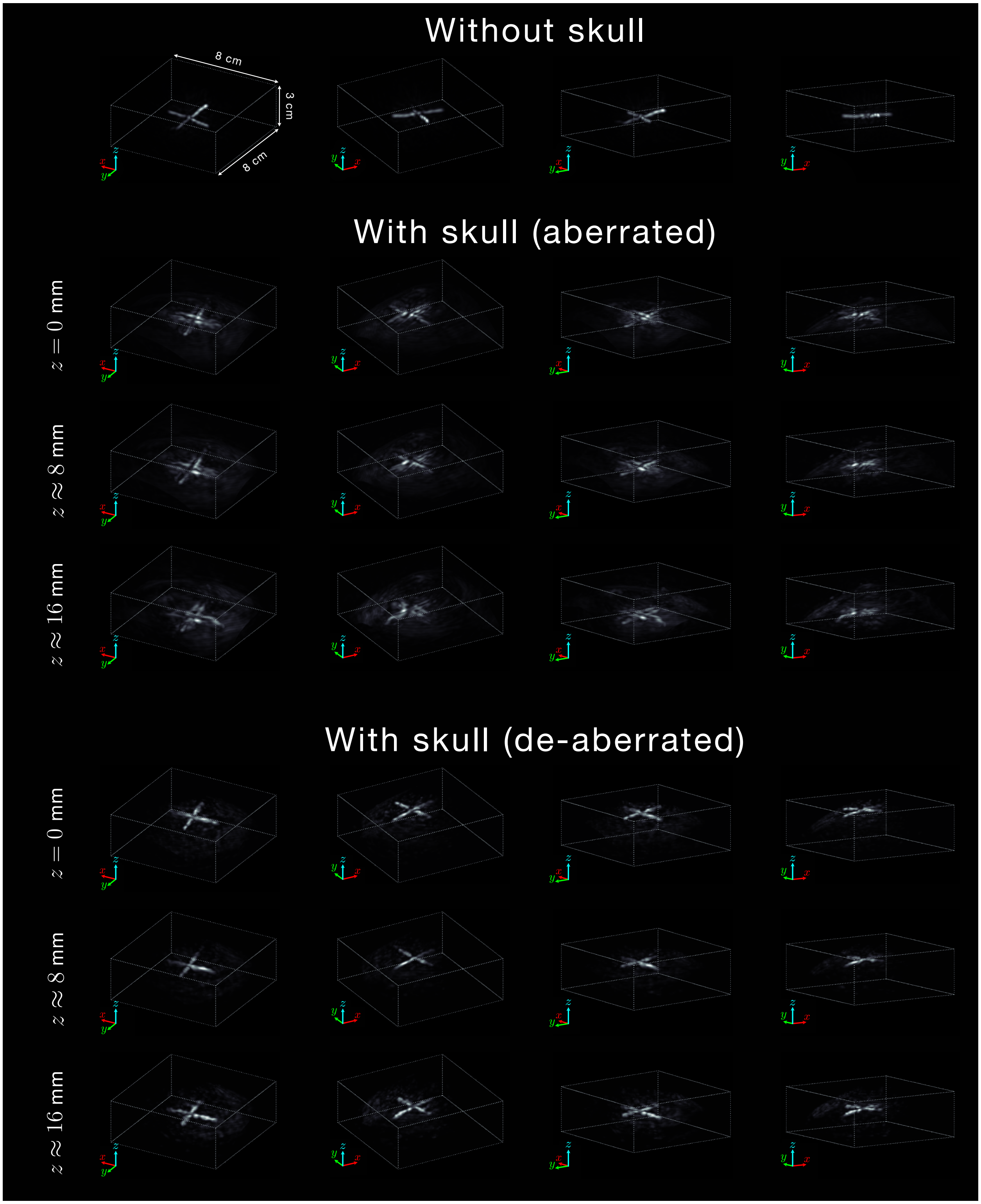

Supplementary Fig. 2 | MAPs of the reconstructed phantom images in Figs. 4c and 4d along different directions to show the 3D structure.

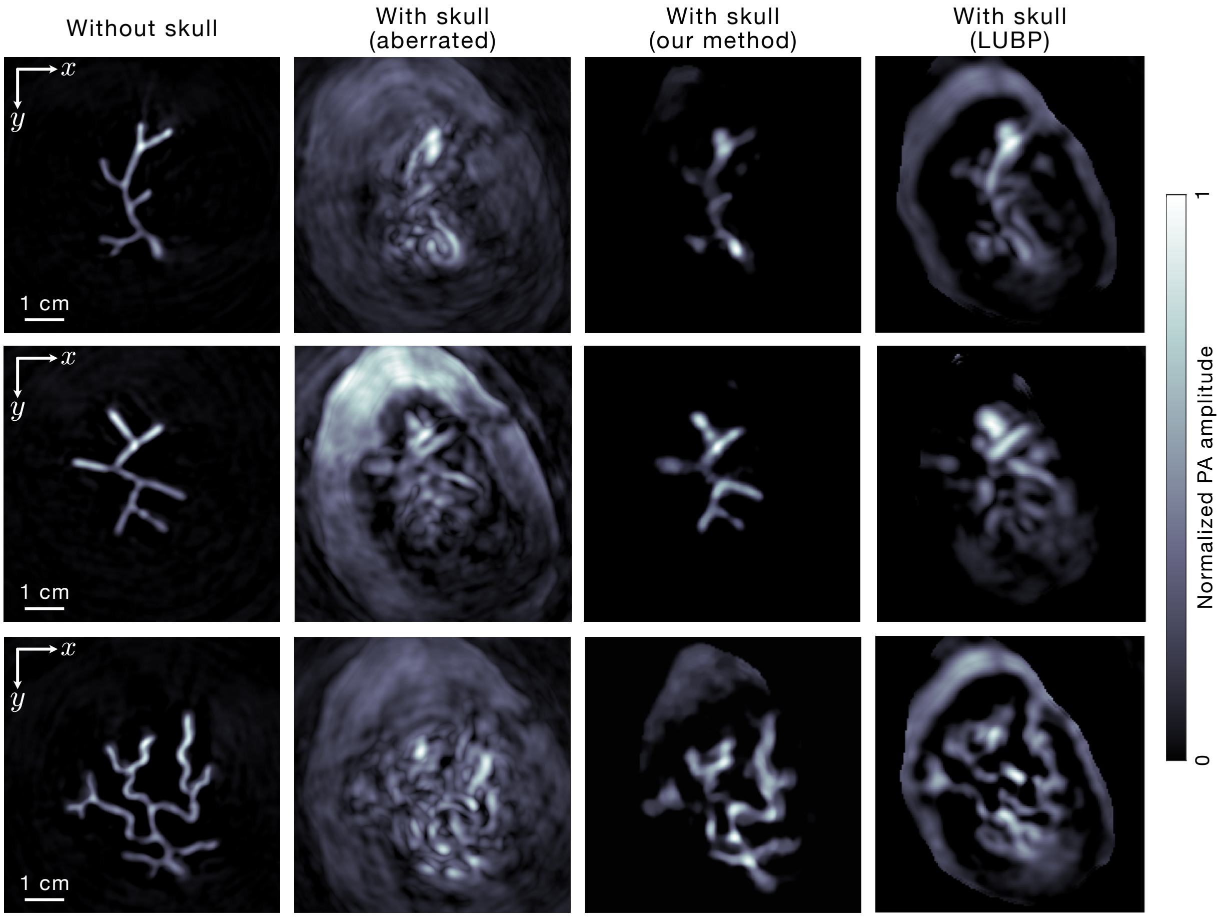

Supplementary Fig. 3 | Comparison of LUBP (1) with our de-aberration method.

Supplementary Methods 1 | Reconstruction algorithm

Algorithm 1 | Transcranial PA image reconstruction

Input: $\boldsymbol{v}_p$ (compression speed map), $\boldsymbol{v}_s$ (shear speed map), $\boldsymbol{\rho}$ (density map), $\boldsymbol{d}$ (measured data), $\boldsymbol{r}_s$ (transducer coordinates), K (max. iterations), $\lambda_{\text{TV}} > 0$ (TV weight), $\lambda_{\ell_1} > 0$ (ℓ_1 weight)

Output: $\hat{\boldsymbol{p}}_0$ (reconstructed image)

$\boldsymbol{d} \leftarrow \text{LowPassFilter}(\boldsymbol{d}, \text{cutoff} = 0.5\,\text{MHz})$ ▷ 2nd-order Butterworth filter
$\mathcal{A}(\cdot) \triangleq \text{ForwardOp}(\cdot)$ ▷ Define forward operator
$\mathcal{A}^\dagger(\cdot) \triangleq \text{AdjointOp}(\cdot)$ ▷ Define adjoint operator
$L \leftarrow \text{PowerIteration}(\mathcal{A}, \mathcal{A}^\dagger, \text{tol} = 10^{-2}, \text{maxIter} = 10)$ ▷ Estimate Lipschitz constant
$\mu \leftarrow 1/L$ ▷ Set step size
$\boldsymbol{x}_0 \leftarrow \boldsymbol{0},\ \boldsymbol{y}_1 \leftarrow \boldsymbol{x}_0$ ▷ Initialize variables
for $k = 1, 2, \ldots, K$ **do**
    $\boldsymbol{x}_k \leftarrow \boldsymbol{y}_k - \mu \mathcal{A}^\dagger(\mathcal{A}\,\boldsymbol{y}_k - \boldsymbol{d})$ ▷ Gradient descent step
    $\boldsymbol{x}_k \leftarrow \text{ProxTV}(\boldsymbol{x}_k, \mu\lambda_{\text{TV}})$ ▷ TV regularization
    $\boldsymbol{x}_k \leftarrow \max(\boldsymbol{x}_k - \mu\lambda_{\ell_1}, \boldsymbol{0})$ ▷ ℓ_1 regularization and positivity
    $\beta_k \leftarrow \frac{k-1}{k+2}$ ▷ Update momentum parameter
    $\boldsymbol{y}_{k+1} \leftarrow \boldsymbol{x}_k + \beta_k(\boldsymbol{x}_k - \boldsymbol{x}_{k-1})$ ▷ Momentum step
$\hat{\boldsymbol{p}}_0 \leftarrow \boldsymbol{x}_K$

function $\text{ForwardOp}(\boldsymbol{x})$ ▷ $\mathcal{A} = SA$: Forward operator (uses global $\boldsymbol{v}_p, \boldsymbol{v}_s, \boldsymbol{\rho}, \boldsymbol{r}_s$)
    $\boldsymbol{u} \leftarrow \text{WavePropagate}(\boldsymbol{x}, \boldsymbol{v}_p, \boldsymbol{v}_s, \boldsymbol{\rho})$
    return $\text{Sampling}(\boldsymbol{u}, \boldsymbol{r}_s)$

function $\text{AdjointOp}(\boldsymbol{y})$ ▷ $\mathcal{A}^\dagger = A^\dagger S^\dagger$: Adjoint operator
    $\boldsymbol{u} \leftarrow \text{AdjointSampling}(\boldsymbol{y}, \boldsymbol{r}_s)$
    return $\text{BackProject}(\boldsymbol{u}, \boldsymbol{v}_p, \boldsymbol{v}_s, \boldsymbol{\rho})$

function $\text{ProxTV}(\boldsymbol{z}, \lambda)$ ▷ TV proximal operator (e.g., solved iteratively via ADMM)
    return $\arg\min_{\boldsymbol{x}} \frac{1}{2}\|\boldsymbol{x} - \boldsymbol{z}\|_2^2 + \lambda\|\nabla\boldsymbol{x}\|_1$

function $\text{PowerIteration}(\mathcal{B}, \mathcal{B}^\dagger, \text{tol}, \text{maxIter})$ ▷ Estimate Lipschitz constant $L = \sigma_{\max}^2(\mathcal{B})$
    $\boldsymbol{v} \leftarrow$ random vector of appropriate length ▷ Initialize random vector
    $\boldsymbol{v} \leftarrow \boldsymbol{v}/\|\boldsymbol{v}\|_2$ ▷ Normalize
    $\lambda_{\text{prev}} \leftarrow 0$
    for $i = 1, 2, \ldots, \text{maxIter}$ **do**
        $\boldsymbol{u} \leftarrow \mathcal{B}(\boldsymbol{v})$ ▷ Forward operation
        $\boldsymbol{w} \leftarrow \mathcal{B}^\dagger(\boldsymbol{u})$ ▷ Adjoint operation
        $\lambda_{\text{curr}} \leftarrow \|\boldsymbol{w}\|_2$ ▷ Estimate $\sigma_{\max}^2$ (eigenvalue of $\mathcal{B}^\dagger\mathcal{B}$)
        $\boldsymbol{v} \leftarrow \boldsymbol{w}/\lambda_{\text{curr}}$ ▷ Normalize
        if $|\lambda_{\text{curr}} - \lambda_{\text{prev}}| < \text{tol} \cdot \lambda_{\text{curr}}$ **then**
            break
        $\lambda_{\text{prev}} \leftarrow \lambda_{\text{curr}}$
    return λ_{curr} ▷ Return Lipschitz constant L

Supplementary References

1. S. Na, X. Yuan, L. Lin, J. Isla, D. Garrett, L. V. Wang, Transcranial photoacoustic computed tomography based on a layered back-projection method. *Photoacoustics* **20**, 100213 (2020).
